# Effects of Sleeve Gastrectomy and Treadmill Exercise on Skeletal Muscle and Ectopic Fat in High-Fat Diet-Induced Obese Rats

**DOI:** 10.3390/ijms26115294

**Published:** 2025-05-30

**Authors:** Takaaki Noguchi, Yuichi Yoshida, Koro Gotoh, Satoshi Nagai, Kentaro Sada, Naoki Matsuda, Miho Suzuki, Akiko Kudo, Shotaro Miyamoto, Yoshinori Ozeki, Takashi Ozaki, Takeshi Nakata, Akihiro Fukuda, Takayuki Masaki, Hirotaka Shibata

**Affiliations:** 1 Department of Endocrinology, Metabolism, Rheumatology and Nephrology, Faculty of Medicine, Oita University, Yufu 879-5593, Japan; noguchitakaaki@oita-u.ac.jp (T.N.); y-yoshida@oita-u.ac.jp (Y.Y.); n-satoshi@oita-u.ac.jp (S.N.); sadaken@oita-u.ac.jp (K.S.); matsuda20656@oita-u.ac.jp (N.M.); m-suzuki@oita-u.ac.jp (M.S.); kudou3@oita-u.ac.jp (A.K.); shoutarou1029@oita-u.ac.jp (S.M.); ozeki23@oita-u.ac.jp (Y.O.); t-ozaki@oita-u.ac.jp (T.O.); nakata@oita-u.ac.jp (T.N.); akifukuda@oita-u.ac.jp (A.F.); 2 Faculty of Welfare and Health Sciences, Oita University, Oita 870-1192, Japan; 3 Geriatric Nursing, Department of Nursing, Faculty of Medicine, Oita University, Yufu 879-5593, Japan; masaki@oita-u.ac.jp

**Keywords:** adiponectin, exercise, myoblast determination protein 1 (MyoD), peri-muscular adipose tissue (PMAT), sarcopenic obesity, sleeve gastrectomy (SG)

## Abstract

A high-fat diet (HFD) can lead to obesity and skeletal muscle atrophy. Sleeve gastrectomy (SG) improves obesity and increases skeletal muscle mass. This study examined whether SG prevented skeletal muscle atrophy in a diet-induced rat obesity rat model. First, 8-week-old male Sprague-Dawley rats underwent surgical (sham-operated or SG) and dietary (standard, high-fat diet, or same pair feeding as SG [PF]) interventions without exercise. In the second experiment, treadmill exercise was added for 4 weeks post-SG (SG + Ex). In the third experiment, rats received an adiponectin receptor agonist (AdipoRon) injection. The HFD induced weight gain and decreased muscle fiber area. SG + Ex reversed these levels, followed by increases in adiponectin in the blood and skeletal muscle and myoblast determination protein 1 (MyoD) and decreased peri-muscular adipose tissue (PMAT) mass, but SG alone did not. No similar changes were observed in the PF group, with or without exercise. Injection of AdipoRon had a similar effect on skeletal muscle and PMAT as SG + Ex. The combination of SG and exercise, but not calorie restriction alone, had better impacts on skeletal muscle and PMAT than SG or exercise alone.

## 1. Introduction

Obesity is rising globally [1], and is linked with diabetes, coronary artery disease, malignant tumors, and sleep apnea syndrome [2]. Furthermore, it has been reported that obesity can cause excess fat accumulation in skeletal muscles, inflammation, and a decline in sex hormones and growth hormone which negatively affect skeletal muscles, leading to sarcopenia, which is characterized by a decrease in muscle mass and strength [3]. The coexistence of excess fat accumulation with decreased muscle mass and function is defined as sarcopenic obesity [4], which reportedly increases the risk of cognitive dysfunction [5], bone fractures [6], and mortality [7].

Sleeve gastrectomy (SG) is the most commonly performed type of bariatric surgery procedure worldwide [8]. SG improves diabetes [9] and hypertension [10]. We reported that the use of SG in obese patients increased skeletal muscle mass per body weight using bioelectrical impedance analysis [11], and that this effect continued three years later [12]. While SG effectively reduces body weight, it has been reported that the risk of developing sarcopenia is increased due to decreased physical activity after SG [13]. The preservation of skeletal muscle mass is crucial, but the current literature lacks studies investigating strategies to mitigate muscle mass loss after SG. Exercise is known to promote muscle hypertrophy and improve metabolic health, but its effect on muscle mass preservation in combination with SG has not been well studied. Very few animal studies on sarcopenic obesity and SG have shown that SG, exercise, and appropriate nutritional supplementation improve muscle fibers and muscle contractile force by increasing insulin-like growth factor 1 (IGF-1) and myogenin [14]. However, in this study, SG without exercise was associated with skeletal muscle atrophy and muscle weakness owing to increased inflammatory and oxidative markers; some clinical studies have reported a decrease in skeletal muscle mass after SG and an increased risk of sarcopenia [13].

Adiponectin, one of the adipokines secreted by adipose tissue, is reportedly lower in obesity than in non-obese subjects [15]. It has been reported that SG increases serum adiponectin level [16], and adiponectin increases the myogenic transcription factor myoblast determining protein 1 (MyoD), thereby increasing skeletal muscle size [17]. Therefore, we hypothesized the possibility that SG could improve sarcopenic obesity by increasing MyoD expression in the skeletal muscle by increasing adiponectin levels in a diet-induced obese rat model.

This study aims to evaluate the effects of SG and exercise on body weight, skeletal muscle, and adipose tissue in obese rat models.

## 2. Results

### 2.1. Baseline Body Weight

In Experiments I, II, and III, there were no significant differences in baseline body weight between the groups (Table 1, Table 2 and Table 3).

### 2.2. Experiment × Time Interaction Effects on Body Weight

First, a two-way analysis of variance (ANOVA) was performed to evaluate the effects of the between-subjects factor Experiments I–III and the within-subjects factor time (before intervention, 4 weeks after intervention) on body weight. The means and standard deviations for body weight are presented in Table 4.

The results indicated a significant main effect for the between-subjects factor Experiments I–III, F(2, 60) = 1.005, *p* = 0.372, partial η^2^ = 0.032; a significant main effect for the within-subjects factor time, F(1, 60) = 16.384, *p* < 0.001, partial η^2^ = 0.214; and a significant interaction between the between-subjects factor Experiments I–III and the within-subjects factor time, F(2, 60) = 18.490, *p* < 0.001, partial η^2^ = 0.381. Simple main effects tests indicated that body weight 4 weeks after the intervention was significantly lower than before the intervention (*p* < 0.001).

Second, a two-way ANOVA was performed to evaluate the effects of the between-subjects factor Experiment I and the within-subjects factor time on body weight. The means and standard deviations for body weight are presented in Table 5.

The results indicated a significant main effect for the between-subjects factor Experiment I, F(3, 20) = 4.190, *p* = 0.019, partial η^2^ = 0.386; a significant main effect for the within-subjects factor time, F(1, 20) = 6.386, *p* = 0.020, partial η^2^ = 0.242; and a significant interaction between the between-subjects factor Experiment I and the within-subjects factor time, F(3, 20) = 8.119, *p* < 0.001, partial η^2^ = 0.549. Simple main effects tests indicated that body weight 4 weeks after the intervention was significantly lower than before the intervention (*p* = 0.020).

Third, a two-way ANOVA was performed to evaluate the effects of the between-subjects factor Experiment I and the within-subjects factor time on body weight. The means and standard deviations for body weight are presented in Table 6.

The results indicated a significant main effect for the between-subjects factor Experiment *II*, F(3, 20) = 14.695, *p* < 0.001, partial η^2^ = 0.688; a significant main effect for the within-subjects factor time, F(1, 20) = 85.823, *p* < 0.001, partial η^2^ = 0.811; and a significant interaction between the between-subjects factor Experiment *II* and the within-subjects factor time, F(3, 20) = 6.132, *p* = 0.004, partial η^2^ = 0.479. Simple main effects tests indicated that body weight 4 weeks after the intervention was significantly lower than before the intervention (*p* < 0.001).

Fourth, a two-way ANOVA was performed to evaluate the effects of the between-subjects factor Experiment I and the within-subjects factor time on body weight. The means and standard deviations for body weight are presented in Table 7.

The results indicated a significant main effect for the between-subjects factor Experiment III, F(2, 12) = 5.951, *p* = 0.016, partial η^2^ = 0.498; a significant main effect for the within-subjects factor time, F(1, 12) = 20.311, *p* < 0.001, partial η^2^ = 0.629; and a significant interaction between the between-subjects factor Experiment *III* and the within-subjects factor time, F(2, 12) = 4.392, *p* = 0.037, partial η^2^ = 0.423. Simple main effects tests indicated that body weight 4 weeks after the intervention was significantly lower than before the intervention (*p* < 0.001).

### 2.3. Effects of SG Without Exercise on Body Weight and Food Intake

**Figure 1:**
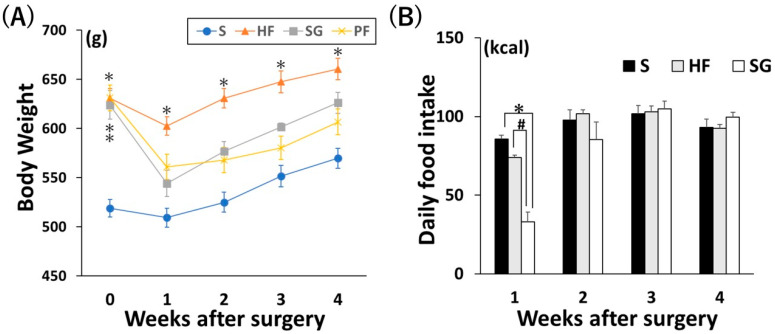
Effects of SG without exercise on body weight and food intake. (**A**) Change in body weight after surgery. (**B**) Daily food intake after surgery. * *p* < 0.05 versus S, # *p* < 0.05 versus HF. Data are mean values ± SEM. S, standard diet-fed rats subjected to sham surgery; HF, high-fat diet-fed rats subjected to sham surgery; SG, high-fat diet-fed rats subjected to sleeve gastrectomy; PF, pair-fed rats subjected to sham surgery.

Before surgery, the SG and pair-feeding (PF) groups had significantly higher body weights than the standard diet-fed (S) group. However, 4 weeks after surgery, the body weights of the SG and PF groups were equivalent to those of the S group (Figure 1A). Daily caloric intake was significantly lower in the SG group than in the S and high-fat diet-fed (HF) groups one week post-surgery (Figure 1B, *p* < 0.05). This decrease was most pronounced during the first week after SG, and no significant change was observed two weeks after surgery.

### 2.4. Muscle Changes Caused by SG Without Exercise

**Figure 2:**
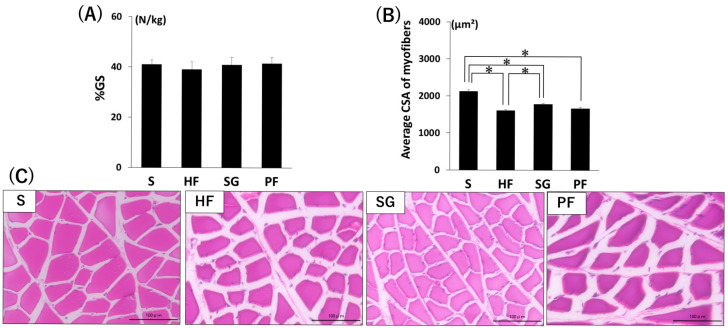
Muscle changes caused by SG without exercise. (**A**) %GS. (**B**) Mean cross-sectional area of gastrocnemius muscle fibers. (**C**) Representative images of H & E-stained gastrocnemius muscle selections. Bars = 100 µm. * *p* < 0.05. Data are mean values ± SEM. S, standard diet-fed rats subjected to sham surgery; HF, high-fat diet-fed rats subjected to sham surgery; SG, high-fat diet-fed rats subjected to sleeve gastrectomy; PF, pair-fed rats subjected to sham surgery; %GS, grip strength per body weight; CSA, cross-sectional area.

Although there was no significant difference in the grip strength per body weight (%GS) after surgery between any of the groups, the HF group tended to have a lower %GS than the other groups did (Figure 2A). The muscle fiber area was significantly increased in the SG group compared with the in the HF group, but was significantly lower in the HF, SG, and PF groups than in the S group (Figure 2B,C, *p* < 0.05).

### 2.5. Muscle-Related Protein Levels and PMAT Measurement of SG Without Exercise

**Figure 3:**
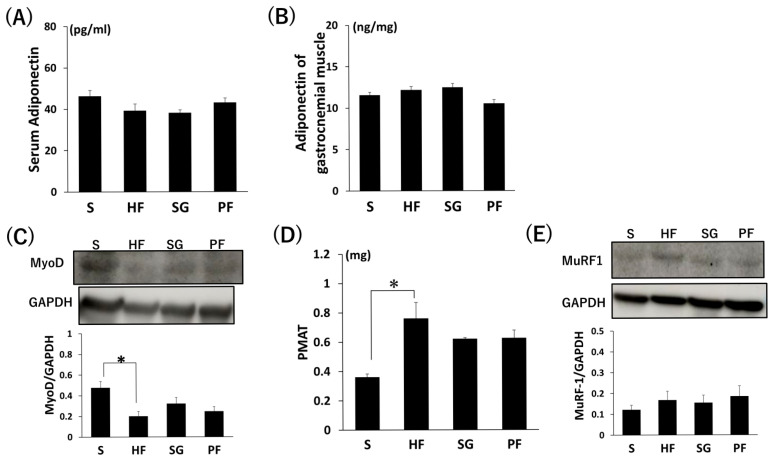
Muscle-related protein levels and PMAT measurement of SG without exercise. (**A**,**B**) Serum and gastrocnemius muscle adiponectin protein levels were determined by ELISA. (**C**) MyoD protein levels were determined by Western blotting. (**D**) PMAT. (**E**) MuRF-1 protein levels were determined by Western blotting. * *p* < 0.05. Data are mean values ± SEM. S, standard diet-fed rats subjected to sham surgery; HF, high-fat diet-fed rats subjected to sham surgery; SG, high-fat diet-fed rats subjected to sleeve gastrectomy; PF, pair-fed rats subjected to sham surgery; PMAT, peri-muscular adipose tissue.

There were no significant differences in the adiponectin protein levels in the serum and gastrocnemius muscle among the four groups (Figure 3A,B). The protein levels of MyoD in the gastrocnemius muscle were significantly lower in the HF group compared to the S group (Figure 3C, *p* < 0.05). Peri-muscular adipose tissue (PMAT) was significantly higher in the HF group compared to the S group (Figure 3D, *p* < 0.05). There were no significant differences in the protein levels of muscle ring finger 1 (MuRF-1) in the gastrocnemius muscle among the four groups (Figure 3E).

### 2.6. Effects of Combined Sleeve Gastrectomy and Treadmill Exercise on Body Weight and Food Intake

**Figure 4:**
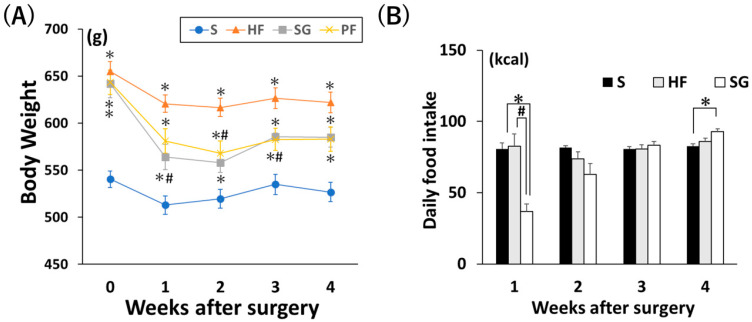
Effects of SG and exercise on body weight and food intake. (**A**) Change in body weight after surgery. (**B**) Daily food intake after surgery. * *p* < 0.05 versus S, # *p* < 0.05 versus HF. Data are mean values ± SEM. S, standard diet-fed rats subjected to sham surgery; HF, high-fat diet-fed rats subjected to sham surgery; SG, high-fat diet-fed rats subjected to sleeve gastrectomy; PF, pair-fed rats subjected to sham surgery.

Although the SG and PF groups temporarily lost weight after surgery, the body weights of the SG and PF groups were greater than those of the S group at 4 weeks after surgery (Figure 4A). Daily caloric intake was significantly lower in the SG group than in the S and HF groups one week after surgery (Figure 4B, *p* < 0.05). This decrease was most pronounced during the first week after SG, and no significant change was observed two weeks after surgery.

### 2.7. Muscle Changes Caused by SG and Exercise

**Figure 5:**
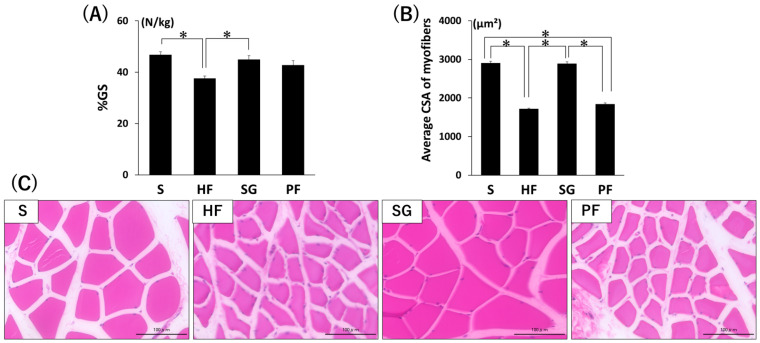
Muscle changes caused by SG and exercise. (**A**) %GS. (**B**) Mean cross-sectional area of gastrocnemius muscle fibers. (**C**) Representative images of H & E-stained gastrocnemius muscle selections. Bars = 100 µm. * *p* < 0.05. Data are mean values ± SEM. S, standard diet-fed rats subjected to sham surgery; HF, high-fat diet-fed rats subjected to sham surgery; SG, high-fat diet-fed rats subjected to sleeve gastrectomy; PF, pair-fed rats subjected to sham surgery; %GS, grip strength per body weight; CSA, cross-sectional area.

When comparing %GS, there was no significant difference between the HF and PF groups and between the SG and PF groups; however, the SG group was significantly higher than the HF group (Figure 5A). The muscle fiber area was significantly higher in the S and SG groups than in the HF and PF groups (Figure 5B,C, *p* < 0.05).

### 2.8. Muscle-Related Protein Levels and PMAT Measurement of SG and Exercise

**Figure 6:**
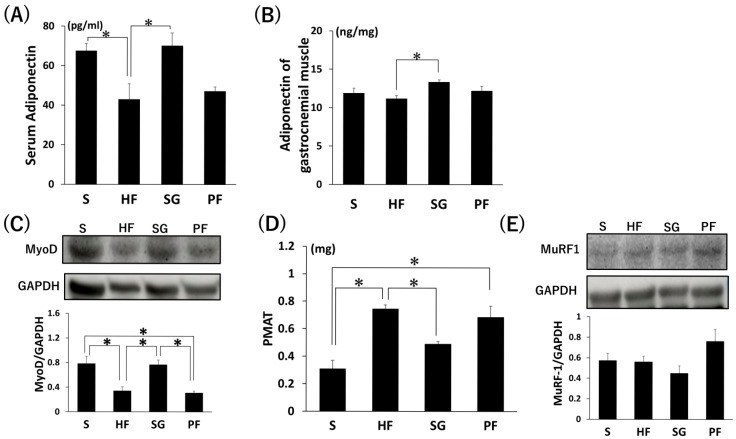
Muscle-related protein levels and PMAT measurement of SG and exercise. (**A**,**B**) Serum and gastrocnemius muscle adiponectin protein levels were determined by ELISA. (**C**) MyoD protein levels were determined by Western blotting. (**D**) PMAT. (**E**) MuRF-1 protein levels were determined by Western blotting. * *p* < 0.05. Data are mean values ± SEM. S, standard diet-fed rats subjected to sham surgery; HF, high-fat diet-fed rats subjected to sham surgery; SG, high-fat diet-fed rats subjected to sleeve gastrectomy; PF, pair-fed rats subjected to sham surgery; PMAT, peri-muscular adipose tissue.

The protein levels of serum adiponectin levels were significantly higher in the S and SG groups than in the HF group (Figure 6A, *p* < 0.05). Adiponectin levels in the gastrocnemius muscle were significantly higher in the SG group than in the HF group (Figure 6B, *p* < 0.05). MyoD protein levels in the gastrocnemius muscle were significantly higher in the S and SG groups than in the HF and PF groups (Figure 6C, *p* < 0.05). PMAT was significantly higher in the HF and PF groups than in the S and SG groups, with the SG group also showing significantly lower than the HF group (Figure 6D, *p* < 0.05). There were no significant differences in the protein levels of MuRF-1 in the gastrocnemius muscle among the four groups (Figure 6E).

### 2.9. Effects of AdipoRon on Body Weight and Food Intake

**Figure 7:**
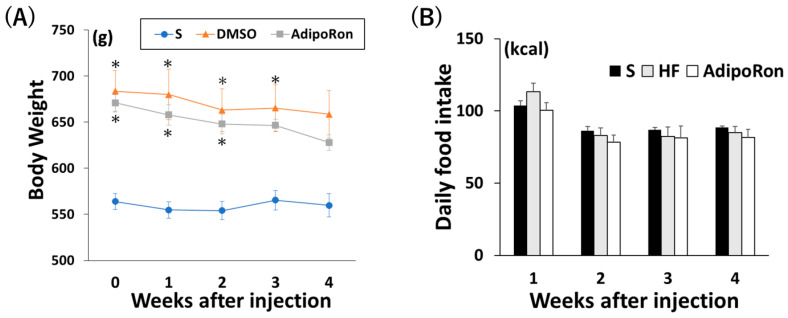
Effects of AdipoRon on body weight and food intake. (**A**) Change in body weight after injection. (**B**) Daily food intake after surgery. * *p* < 0.05 versus S. Data are mean values ± SEM. S, standard diet-fed rats injected with 1% dimethyl sulfoxide (DMSO); HF, high-fat diet-fed rats injected with DMSO; AdipoRon, high-fat diet-fed rats injected with AdipoRon.

Before AdipoRon injection, the high-fat diet (HFD)-fed groups were significantly heavier than the standard diet group. There was no significant difference in body weight between the three groups after 4 weeks of injection (Figure 7A). There were no significant differences in daily calorie intake among the three groups (Figure 7B).

### 2.10. Muscle Changes Caused by AdipoRon

**Figure 8:**
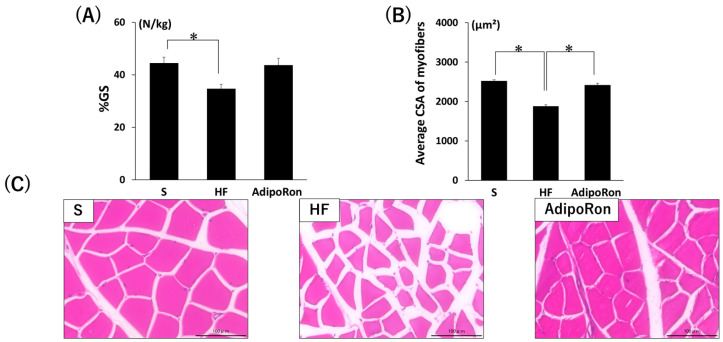
Muscle changes caused by AdipoRon. (**A**) %GS. (**B**) Mean cross-sectional area of gastrocnemius muscle fibers. (**C**) Representative images of H & E-stained gastrocnemius muscle selections. Bars = 100 µm. * *p* < 0.05. Data are mean values ± SEM. S, standard diet-fed rats injected with DMSO; HF, high-fat diet-fed rats injected with DMSO; AdipoRon, high-fat diet-fed rats injected with AdipoRon; %GS, grip strength per body weight; CSA, cross-sectional area.

When comparing the %GS, the HF group showed a significant decrease compared to the S group, but the AdipoRon group showed no significant difference from the S group (Figure 8A). The muscle fiber area was significantly higher in the S and AdipoRon groups than in the HF group (Figure 8B,C, *p* < 0.05).

### 2.11. Muscle-Related Protein Levels and PMAT Mass Measurement of AdipoRon

**Figure 9:**
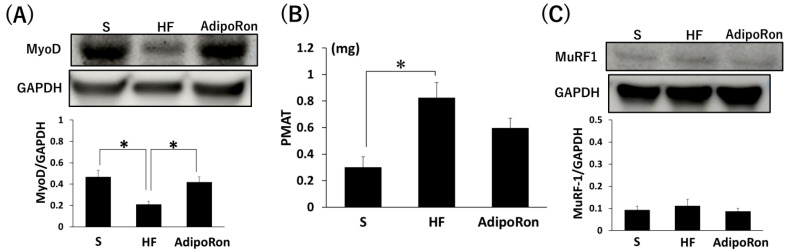
Muscle-related protein levels and PMAT mass measurement of AdipoRon. (**A**) MyoD proteins levels were determined by Western blotting. (**B**) PMAT. (**C**) MuRF-1 protein levels were determined by Western blotting. * *p* < 0.05. Data are mean values ± SEM. S, standard diet-fed rats injected with DMSO; HF, high-fat diet-fed rats injected with DMSO; AdipoRon, high-fat diet-fed rats injected with AdipoRon; PMAT, peri-muscular adipose tissue.

MyoD protein levels in the gastrocnemius muscle were significantly higher in the S and AdipoRon groups than in the HF group (Figure 9A, *p* < 0.05). PMAT was significantly higher in the HF group than in the S group (Figure 9B, *p* < 0.05); however, there was no significant difference between the S and AdipoRon groups and between the HF and AdipoRon groups. There were no significant differences in the protein levels of MuRF-1 in the gastrocnemius muscle among the three groups (Figure 9C).

## 3. Discussion

In this study, we investigated whether SG benefits the skeletal muscles of rats with diet-induced obesity. In rats that underwent exercise after SG, we observed increases in %GS, muscle fiber area, serum and skeletal muscle adiponectin, and skeletal muscle MyoD, and a decrease in PMAT mass. However, these changes were not observed in the SG without exercise. PF rats that underwent exercise after SG did not achieve these beneficial effects on the skeletal muscle. The injection of an adiponectin receptor agonist was effective in the muscle in the absence of body weight reduction, similar to the results of adding exercise after SG. Increased serum adiponectin by the combination of SG and exercise could be one of the physiological mechanisms that promote muscle growth by increasing MyoD expression and improving ectopic fat metabolism.

### 3.1. HFD and Muscle Weakness

Our study showed that HFD-induced obesity was associated with decreased %GS and muscle fiber area. This finding supports previous studies reporting that an HFD is associated with obesity, and causes skeletal muscle atrophy [18]. Fatty acids, diacylglycerol (DAG), and ceramides induce apoptosis and reduce myoblast proliferation and differentiation, possibly via myostatin activation and inhibition of MyoD and myogenin expression and/or activity [19].

### 3.2. The Effect of Adiponectin on Muscle

Our study showed that increased MyoD was associated with elevated serum adiponectin levels. Adiponectin has been reported to induce MyoD expression to promote muscle cell proliferation and differentiation through its receptor in skeletal muscle [17,20]. It also promotes myoblast survival, inhibits apoptosis through an AMP-activated protein kinase (AMPK)-dependent mechanism [21], and promotes muscle differentiation by activating myogenin and myogenic regulatory factor 4 [17], both essential for inducing cell fusion into multinucleated myotubes [22]. In this study, if the increase in skeletal muscle mass was due to an increase in adiponectin levels, it was expected that MyoD would change in a similar manner. Measurement of MyoD revealed that, similar to adiponectin, MyoD decreased with an HFD-induced weight gain, and MyoD levels improved with the combination of SG and exercise. In Experiment III, to confirm that increased serum adiponectin levels enhanced MyoD expression, we injected the adiponectin receptor agonist AdipoRon into obese rats. We observed an increase in MyoD, improved %GS, and reduced muscle atrophy, similar to the effects of the combination of SG and exercise.

### 3.3. The Effect of Adiponectin on PMAT

Our study showed that the combination of SG and exercise significantly reduced PMAT surrounding the gastrocnemius muscles. In addition, in Experiment III, AdipoRon injection reduced the weight of PMAT increased by the HFD, suggesting that increased serum adiponectin reduces ectopic fat. Increased ectopic fat in obese patients has been reported to correlate with decreased adiponectin [23]. Ectopic fat is caused by excess energy intake, leading to lipid redistribution and accumulation of ectopic lipids in lean tissues [24]. It has been shown that PMAT increases in parallel with muscle atrophy and decreases in the size of skeletal muscle fibrocytes [25]. To the best of our knowledge, there have been no reports focusing on ectopic fat in the skeletal muscle in SG and AdipoRon.

### 3.4. Improvement of Muscle Atrophy and PMAT by Combining SG and Exercise

Our study showed that only the combination of SG and treadmill exercise significantly improved grip strength and muscle fiber area, whereas SG without exercise and sham surgery with exercise did not increase grip strength and muscle fiber area. These results suggest that the combination treatment is particularly effective for reversing HFD-induced muscle atrophy. Importantly, the PF group, which received the same amount of food as the SG group, did not show similar improvements, indicating that the changes were not solely due to reduced food intake or weight loss. These results indicate that the combination of SG and treadmill exercise is an effective treatment for muscle weakness induced by an HFD. This change was not observed in the PF group, which had an equivalent food intake to that of the SG group, indicating that changes in food intake and body weight alone did not significantly change the skeletal muscle. In Experiment II, compared to non-obese rats, obese rats showed a significant decrease in serum adiponectin and MyoD protein levels, which were increased to the same levels as non-obese rats by the combination of SG and treadmill exercise. An increase in serum adiponectin levels in obese patients after SG was previously reported by Gomez-Martin et al. [26]. They reported that both SG and Roux-en-Y gastric bypass (RYGB) increased serum adiponectin levels [26], suggesting changes in gastrointestinal hormone levels. However, in this study, adiponectin levels did not increase in the SG without exercise. Additionally, a report by Gomez-Martin et al. showed that SG led to a smaller increase in serum adiponectin levels than RYGB [26]. Another report reported that SG did not increase adiponectin expression in adipose tissue [27], suggesting that the effect of SG on adiponectin levels remains controversial. Other studies have also reported that serum adiponectin levels differ depending on exercise intensity in rats not performing SG [28]. In this study, treadmill exercise after SG increased adiponectin levels, suggesting that this combination treatment is suitable for increasing adiponectin levels.

Based on these findings, this study suggests that combining SG with exercise increases serum and skeletal muscle adiponectin levels, and the subsequent enhancement of MyoD expression improves the reduced %GS and skeletal muscle atrophy induced by obesity due to an HFD. Furthermore, this combination reduced the PMAT. These results indicate that when obese patients undergo SG, muscle strength is likely to improve through postoperative exercise instruction, and metabolic improvements can be expected compared with those who do not engage in exercise. In Japan, obese patients aged 66 years or older are currently not eligible for SG. However, based on the results of this study, it appears that elderly people who are prone to sarcopenic obesity are particularly suitable for treatments that combine SG and exercise.

The question arises as to why and how the combination of SG and exercise, but not SG or exercise alone, has a positive impact on adiponectin and skeletal muscles. A significant reduction in PMAT is likely a key factor; however, the molecular interactions between PMAT and skeletal muscles remain to be elucidated.

### 3.5. Change in the Muscle Atrophy Markers

Our study examined MyoD, which is associated with muscle hypertrophy, and also investigated MuRF-1, a key protein involved in skeletal muscle atrophy [29]. MuRF-1 is one of the major ubiquitin ligases in skeletal muscles. Cachexia factors such as TNFα have been shown to activate the nuclear factor-kappaB (NF-κB) transcriptional pathway, contributing to skeletal muscle atrophy, partly through NF-κB-mediated increased expression of MuRF-1 [30]. It has been reported that an HFD increases the protein expression of MuRF-1, leading to skeletal muscle atrophy [31]. In this study, MuRF-1 levels did not change in response to an HFD, and it could not be reduced by SG or exercise.

### 3.6. Sarcopenic Obesity

Sarcopenic obesity is characterized by the coexistence of muscle weakness and obesity. There is currently no established treatment for sarcopenic obesity. In recent years, glucagon-like peptide-1 receptor agonist (GLP-1RA) and dual glucose-dependent insulinotropic peptide (GIP) and GLP-1 RA have been used as treatments for obesity, but there have been reports raising concerns about muscle weakness due to weight loss [32]. In addition to exercise, testosterone therapy is another approach to increasing muscle mass, but it is associated with various adverse effects [33]. Compared to these treatments, SG and exercise may be a safer and more reliable treatment for preventing sarcopenic obesity.

### 3.7. Strenths and Limitations

This study has several strengths, including the ability to directly compare physiological and molecular outcomes using multiple interventions, including SG, exercise, and AdipoRon, in a controlled experimental setting. We comprehensively evaluated muscle mass, PMAT, and key signaling molecules such as adiponectin and MyoD, providing mechanistic insights into sarcopenic obesity.

However, our study had several limitations. First, this study was only an experiment in a rat model, and because of species differences between rodents and humans, the results may not be generalizable to humans. Second, the results of the treadmill exercise were not necessarily the same as those of all other exercises. The treadmill exercise used in this study was not as intense as in other experiments [34], and the results may vary depending on the exercise intensity. Third, although this was a common obesity model, it did not include a normal diet. Fourth, the combination of SG and exercise, but not either alone, reduced PMAT and increased serum adiponectin levels; however, it remains to be elucidated whether increased serum adiponectin reduces PMAT with a subsequent increase in skeletal muscle increases skeletal muscle with a subsequent decrease in PMAT, or simultaneously reduces PMAT and increases skeletal muscle. Finally, the detailed mechanism by which SG increases adiponectin levels remains unclear, even when compared with previous reports, and this remains a future challenge.

Given these limitations, future studies should incorporate human subjects, include control groups consuming a normal diet, and examine different types and intensities of exercise. Mechanistic investigations using genetic models may further clarify the causal relationships among skeletal muscle, adiponectin, and PMAT. These findings could provide a valuable foundation for developing effective strategies to prevent and treat sarcopenic obesity in clinical settings.

## 4. Materials and Methods

### 4.1. Animals

A total of 63 eight-week-old male Sprague-Dawley (SD) rats were purchased from Jackson Laboratory Japan (Kanagawa, Japan). The rats were housed per cage at a constant temperature (23 ± 1 °C) with 55 ± 10% relative humidity and a 12:12 h light/dark cycle in Oita University Animal Center. The rats had access to food and water ad libitum. All experiments were approved by the Oita University Animal Experimentation Committee and conformed to the Oita University’s Guidelines for Animal Experimentation. The study was conducted in accordance with the ARRIVE guidelines 2.0 (see Files S1 and S2). To assign rats to experimental groups, we used a matched allocation method based on baseline body weight. Rats were divided into four groups based on their body weight. This method was adopted to minimize the possibility of confounding by baseline body weight and to maintain balance between groups. All experimental procedures were performed with alternating group order to avoid assessment bias.

This study employed a between-subjects experimental design to evaluate the effects of SG without exercise, SG plus exercise, and AdipoRon. This study was structured into three separate experiments, each comprising distinct groups and interventions. Additionally, to eliminate the possibility of carryover effects and ensure independent observations, each subject was used in only one experiment.

A priori power analysis was performed to determine the minimum sample size required. Based on pilot data from Experiment II (%GS, *n* = 2–3 per group), the estimated effect size was η^2^ = 0.54. Using a significance level of 0.05 and power of 0.80, the calculated sample size was 4 rats per group (16 total). To ensure robustness against potential data loss, this was increased to 6 rats per group (24 total). Experiment I employed the same sample size as Experiment II (*n* = 6 per group) to ensure statistical power and methodological consistency between experiments and to facilitate the comparison of intervention effects. In Experiment III, the group size was set at five rats per group because of the expected lower risk of mortality compared to SG.

### 4.2. Experiment I: SG Without Exercise

Eight-week-old SD rats were assigned to one of four groups (sample size per group: *n* = 6 in each group, number of groups: 4, total number of subjects: 24 rats). In the S group, rats were fed a standard diet for 10 weeks, followed by a sham operation. After the sham operation, the rats were fed a standard diet for 4 weeks. The remaining rats were fed a HFD for 10 weeks and divided into three groups (HF group, SG group, and PF group). Rats in the HF group were subjected to a sham operation and fed an HFD for another 4 weeks. SG group rats underwent SG and fed an HFD for another 4 weeks. PF group rats underwent a sham operation and were fed the same amount of HFD as SG group rats ad libitum for another 4 weeks. Body weight and food intake were measured daily in all groups (PG3001-S; Mettler-Toledo, Columbus, OH, USA). Food in the PF group was provided ad libitum to the daily intake observed in the SG group. Finally, blood, gastrocnemius muscle, and PMAT of the hind limb samples were collected.

### 4.3. Experiment II: SG Plus Exercise

Eight-week-old SD rats were assigned to one of four groups (sample size per group: *n* = 6 in each group, number of groups: 4, total number of subjects: 24 rats). The four groups were fed the same diet as in Experiment I. After 10 weeks, exercise loading was administered to all the rats using the same diet protocols as in Experiment I for another 4 weeks. Body weight and food intake were measured daily for all groups. The amount of food in the PF group was adapted ad libitum to match the daily intake observed in the SG group. Finally, the blood, gastrocnemius muscle, and PMAT of the hind limb samples were collected.

### 4.4. Experiment III: AdipoRon Injection

Eight-week-old SD rats were assigned to one of three groups (sample size per group: *n* = 5 in each group, number of groups: 3, total number of subjects: 15 rats). In the S group, the rats were fed a standard diet for 10 weeks and then intraperitoneally injected with 1% dimethyl sulfoxide (DMSO) (0.1 mL) twice per week with a standard diet for another 4 weeks. In the HF group, the rats were fed an HFD for 10 weeks, and then DMSO (0.1 mL) was injected intraperitoneally twice a week along with an HFD for another 4 weeks. In the AdipoRon group, the rats were fed an HFD for 10 weeks, followed by an intraperitoneal injection of DMSO with AdipoRon (5 mg/kg) twice a week for another 4 weeks. Finally, the blood, gastrocnemius muscle, and PMAT of the hind limb samples were collected.

### 4.5. SG

The rats were fasted for 24 h before surgery and anesthetized by intraperitoneal injection of medetomidine (Kyoritsu Seiyaku, Tokyo, Japan), midazolam (Nichi-Iko Pharmaceutical, Toyama, Japan), and butorphanol (Meiji Animal Health, Kumamoto, Japan). SGs were performed as previously described [35]. An upper midline abdominal incision was made and the stomach was exposed and exteriorized. Approximately 90% of the forestomach and 70% of the glandular stomach were removed from the greater curvature. For the sham operation, the animals underwent laparotomy and the stomachs were elevated and returned to the abdominal cavity.

### 4.6. Exercise Protocol

The rats were subjected to treadmill exercise at 0° inclination (TM-R-N1, Osaka Microsystems, Osaka, Japan). The training protocol prescribed an exercise frequency of 5 days/week for 4 weeks. The running speed was started at 2 m/min in the first week, increased by 2 m/min every 2 min, and when a maximum speed of 10 m/min was reached, the rats ran at 10 m/min for 20 min. In the second week, the rats ran at 10 m/min for 15 min, rested for 5 min, and ran at 12.5 m/min for 15 min. In the third week, the rats ran at 12.5 m/min for 15 min, rested for 5 min, and then ran at 15 m/min for 15 min. In the fourth week, the rats ran at a speed of 15 m/min for 30 min.

### 4.7. Reagents

AdipoRon (Enzo Life Sciences, Farmingdale, NY, USA), an adiponectin receptor agonist, was dissolved in 1% DMSO (Tokyo Chemical Industry, Tokyo, Japan). The rats received an intraperitoneal injection of 5 mg/kg AdipoRon twice weekly for 4 weeks. The sham group received the same amount of DMSO.

### 4.8. Diets

The standard diet (MFG; Oriental Yeast, Tokyo, Japan) contained 60 kcal% carbohydrates, 26 kcal% protein, and 14 kcal% fat. The HFD (#D12492; Research Diets, New Brunswick, NJ, USA) contained 20 kcal% carbohydrates, 20 kcal% protein, and 60 kcal% fat. Caloric density was 3.55 kcal/g for the standard diet and 5.24 kcal/g for the HFD.

### 4.9. Grip Strength Test

Forelimb grip strength was measured using an electronic grip strength meter (FG-5005; Mother Tool, Nagano, Japan). The rats gripped the grid with their forelimbs and were gently tugged until the grip was released. The tests were performed 4 weeks after surgery or injection. The measurements were repeated twice and the peak force was recorded, normalized to body weight (%GS).

### 4.10. PMAT

The PMAT has been previously reported to exist in the space surrounding the hind limb skeletal muscles [25]. We removed the fat mass and measured the PMAT weight after surgery or medication administration.

### 4.11. Immunohistochemistry

To assess pathological changes in the gastrocnemius muscle, tissues were obtained and fixed in 4% paraformaldehyde. Slices (10 μm thick) from the gastrocnemius muscle were prepared and subsequently stained with hematoxylin and eosin (H & E) by Lintec Corporation, Oita Laboratory (Oita, Japan), a certified histopathology service provider, following standardized protocols to ensure consistent staining quality. The cross-sectional area (CSA) of muscle fibers was examined using an electron microscope (BZ-9000; KEYENCE, Osaka, Japan). The CSA of 100 muscle fibers/animal was randomly measured. Average areas of the muscle fibers in each image were measured using the ImageJ software (version 1.43u; National Institutes of Health, Bethesda, MD, USA). The measurement process involved calibrating the scale based on the microscope’s magnification, outlining the perimeter of each muscle fiber, and calculating the CSA using the ImageJ software.

### 4.12. Western Blot Analysis

Frozen gastrocnemius muscle samples were homogenized in a radioimmunoprecipitation assay (RIPA) buffer, centrifuged, and boiled. The total protein concentration was quantified by Bradford protein assay, and 10 µg of total protein per sample was separated with a 4–20% Mini-Protean TGX gel (Bio-Rad Laboratories, Hercules, CA, USA), and then transferred to a polyvinylidene difluoride (PVDF) membrane (Bio-Rad Laboratories). The primary antibodies used included MyoD (Santa Cruz Biotechnology, Dallas, TX, USA), MuRF-1 (ECM Biosciences, Versailles, KY, USA), and Glyceraldehyde-3-phosphate dehydrogenase (GAPDH) (Abcam plc, Cambridge, UK). Secondary antibody conjugated to horseradish peroxidase (Cytiva, Marlborough, MA, USA) was later added to the membrane, and immunoreactive bands were detected by enhanced chemiluminescence (ImageQuant 800, Cytiva) and quantified using ImageJ. MyoD, MuRF-1 and GAPDH were measured in each specimen, and each protein level was determined as a ratio to GAPDH between groups.

### 4.13. Biochemical Enzyme-Linked Immuno-Sorbent Assay (ELISA) Tests

Adiponectin concentrations in rat serum and gastrocnemius muscle were measured using ELISA kits (Proteintech Group, Rosemont, IL, USA).

### 4.14. Statistical Analysis

Statistical analyses were performed using Statistical Package for the Social Sciences (SPSS) version 29.0.2 for Windows (International Business Machines, Armonk, NY, USA). All data are expressed as means ± SEM. Two-way ANOVA to examine the time × group interaction to determine whether pre-to-post changes in body weight within groups differed across groups. The simple main effect of time was then analyzed to assess within-group changes. Following the two-way ANOVA result, Bonferroni’s post hoc tests as contrast analysis were conducted to estimate the extent to which mean changes differed between group comparison pairs. When reporting descriptive statistics according to the American Psychological Association (APA) style, standard deviation (SD) was used. In addition, one-way ANOVA was used to compare between groups at each time point, and if significant differences were found, post hoc comparisons were performed using Tukey’s honestly significant difference test.

## 5. Conclusions

SG without exercise had little effect on skeletal muscle mass, but the combination of treadmill exercise after SG enhanced serum and skeletal adiponectin levels, followed by increased MyoD expression in skeletal muscle. This results in an increase in %GS and skeletal muscle mass and a decrease in PMAT amount. The combination of SG and exercise may be effective in the treatment of sarcopenic obesity.

These results suggest that the combination of SG and exercise may not only increase muscle mass and reduce ectopic fat, but also address the underlying mechanisms of sarcopenic obesity through increasing adiponectin. This combined intervention may serve as an effective therapeutic strategy for managing sarcopenic obesity.

## Figures and Tables

**Table 1:** Baseline data of each group before the beginning of Experiment I.

Experiment I	S	HF	SG	PF	*p* Value
Body weight (g)	279.6 ± 3.5	278.1 ± 3.3	280.7 ± 1.7	278.7 ± 3.3	N.S.

S, standard diet-fed rats subjected to sham surgery; HF, high-fat diet-fed rats subjected to sham surgery; SG, high-fat diet-fed rats subjected to sleeve gastrectomy; PF, pair-fed rats subjected to sham surgery. The data are expressed as mean ± SEM. N.S., no significance.

**Table 2:** Baseline data of each group before the beginning of Experiment II.

Experiment II	S	HF	SG	PF	*p* Value
Body weight (g)	274.0 ± 3.4	280.0 ± 3.3	276.5 ± 3.6	277.8 ± 1.8	N.S.

S, standard diet-fed rats subjected to sham surgery; HF, high-fat diet-fed rats subjected to sham surgery; SG, high-fat diet-fed rats subjected to sleeve gastrectomy; PF, pair-fed rats subjected to sham surgery. The data are expressed as mean ± SEM. N.S., no significance.

**Table 3:** Baseline data of each group before the beginning of Experiment III.

Experiment III	S	HF	AdipoRon	*p* Value
Body weight (g)	252.8 ± 4.0	253.9 ± 2.0	252.5 ± 2.6	N.S.

S, standard diet-fed rats injected with DMSO; HF, high-fat diet-fed rats injected with DMSO; AdipoRon, high-fat diet-fed rats injected with AdipoRon. The data are expressed as mean ± SEM. N.S., no significance.

**Table 4:** Descriptive statistics of body weight (g) for Experiments I, II, and III.

Intervention	Experiment	Mean	SD
Before	I	601.2	68.8
	II	620.2	54.7
	III	639.5	67.9
4 weeks after	I	615.8	60.1
	II	579.2	43.8
	III	615.4	68.1

SD, standard deviation.

**Table 5:** Descriptive statistics of body weight (g) for Experiment I.

Intervention	Group	Mean	SD
Before	S	519.0	60.1
	HF	630.6	56.8
	SG	624.1	22.7
	PF	631.2	50.3
4 weeks after	S	569.8	58.1
	HF	660.6	67.0
	SG	626.1	25.4
	PF	606.7	40.5

S, standard diet-fed rats subjected to sham surgery; HF, high-fat diet-fed rats subjected to sham surgery; SG, high-fat diet-fed rats subjected to sleeve gastrectomy; PF, pair-fed rats subjected to sham surgery; SD, standard deviation.

**Table 6:** Descriptive statistics of body weight (g) for Experiment II.

Intervention	Group	Mean	SD
Before	S	540.4	21.7
	HF	655.0	25.2
	SG	641.8	35.7
	PF	643.7	31.9
4 weeks after	S	526.8	25.1
	HF	622.0	26.7
	SG	585.1	26.1
	PF	583.1	32.2

S, standard diet-fed rats subjected to sham surgery; HF, high-fat diet-fed rats subjected to sham surgery; SG, high-fat diet-fed rats subjected to sleeve gastrectomy; PF, pair-fed rats subjected to sham surgery; SD, standard deviation.

**Table 7:** Descriptive statistics of body weight (g) for Experiment III.

Intervention	Group	Mean	SD
Before	S	564.0	19.5
	HF	683.5	59.5
	AdipoRon	670.9	35.8
4 weeks after	S	559.7	28.3
	HF	658.5	68.6
	AdipoRon	627.9	57.2

S, standard diet-fed rats injected with DMSO; HF, high-fat diet-fed rats injected with DMSO; AdipoRon, high-fat diet-fed rats injected with AdipoRon; SD, standard deviation.

## Data Availability

The data that support the findings of this study are available from the corresponding author upon reasonable request.

## Supplementary Materials

The following supporting information can be downloaded at: https://www.mdpi.com/article/10.3390/ijms26115294/s1.
