# Supplementary material for: Effects of Sleeve Gastrectomy and Treadmill Exercise on Skeletal Muscle and Ectopic Fat in High-Fat Diet-Induced Obese Rats
Source: Int J Mol Sci. 2025 May 30;26(11):5294. doi: 10.3390/ijms26115294 (PMC12154429; doi:10.3390/ijms26115294)
Supplement: Supplementary file 1 [file ijms-26-05294-s001.zip › File_S2_Additional_ARRIVE_guidelines_2.0_author_checklist_Details_IJMS-3622100.pdf]

ARRIVE Guidelines 2.0 Checklist Supplement

Animal care and monitoring-16a.

A combination of medetomidine, midazolam, and butorphanol was used to induce general anesthesia during all surgical procedures. Postoperative analgesia was not administered. All animals were carefully monitored during the postoperative period. No animals showed signs of pain or distress based on daily clinical observations. The absence of postoperative analgesia is acknowledged as a limitation of the study.

Animal care and monitoring-16b.

No adverse events or unexpected deaths were observed in any of the animals included in the reported experiments. All animals completed the study protocol without requiring early euthanasia or intervention.

Animal care and monitoring-16c.

No specific humane endpoints were predefined in the study. All animals remained in good health throughout the experimental period, and none met criteria requiring early euthanasia.
